# *de novo* Blood Biomarkers in Autism: Autoantibodies against Neuronal and Glial Proteins

**DOI:** 10.3390/bs9050047

**Published:** 2019-04-29

**Authors:** Mohamed B. Abou-Donia, Hagir B. Suliman, Dario Siniscalco, Nicola Antonucci, Passent ElKafrawy, Mulugu V. Brahmajothi

**Affiliations:** 1 Department of Pharmacology and Cancer Biology, Duke University Medical Center, Durham, NC 27710, USA; brahma@duke.edu; 2 Department of Neurobiology, Duke University Medical Center, Durham, NC 27710, USA; 3 Hyperbaric Division, Department of Anesthesiology, Duke University Medical Center, Durham, NC 27710, USA; hagir.suliman@duke.edu; 4 Department of Experimental Medicine, University of Campania, 80138 Naples, Italy; dariosin@uab.edu; 5 Biomedical Center for Autism Research and Treatment, 70124 Bari, Italy; info@antonucci.eu; 6 Faculty of Science, Menofia University, Shebien El-Koom 32615, Egypt; basant.elkafrawi@science.menofia.edu.eg

**Keywords:** autism spectrum disorder, control children, neuronal autoantibodies, autoimmune disorder, maternal autoantibodies, neuronal and astroglial biomarkers

## Abstract

Autism spectrum disorders (ASDs) are the most common neurodevelopmental disorders with unidentified etiology. The behavioral manifestations of ASD may be a consequence of genetic and/or environmental pathology in neurodevelopmental processes. In this limited study, we assayed autoantibodies to a panel of vital neuronal and glial proteins in the sera of 40 subjects (10 children with ASD and their mothers along with 10 healthy controls, age-matched children and their mothers). Serum samples were screened using Western Blot analysis to measure immunoglobulin (IgG) reactivity against a panel of 9 neuronal proteins commonly associated with neuronal degeneration: neurofilament triplet proteins (NFP), tubulin, microtubule-associated proteins (tau), microtubule-associated protein-2 (MAP-2), myelin basic protein (MBP), myelin-associated glycoprotein (MAG), α-synuclein (SNCA) and astrocytes proteins such as glial fibrillary acidic protein (GFAP) and S100B protein. Our data show that the levels of circulating IgG class autoantibodies against the nine proteins were significantly elevated in ASD children. Mothers of ASD children exhibited increased levels of autoantibodies against all panel of tested proteins except for S100B and tubulin compared to age-matched healthy control children and their mothers. Control children and their mothers showed low and insignificant levels of autoantibodies to neuronal and glial proteins. These results strongly support the importance of anti-neuronal and glial protein autoantibodies biomarker in screening for ASD children and further confirm the importance of the involvement of the maternal immune system as an index that should be considered in fetal *in utero* environmental exposures. More studies are needed using larger cohort to verify these results and understand the importance of the presence of such autoantibodies in children with autism and their mothers, both as biomarkers and their role in the mechanism of action of autism and perhaps in its treatment.

## 1. Introduction

Autism spectrum disorder (ASD) is a neurodevelopmental disorder characterized by impaired social interaction, challenges in communication, social isolation “loneliness” and repetitive behaviors “sameness” [1]. Diagnosis is often made as early as 18 months of age, but most patients are not formally diagnosed until they are 5 years old [2]. Children with ASD may also have other disorders including, intellectual disability [3], epilepsy [4], Tourette’s syndrome, difficulty sleeping [5] and many suffer gastrointestinal dysfunctions [6].

Because of the lack of biochemical diagnostic tests, it is unclear whether the symptoms of ASD are originated from diverse etiologies with different manifestations of the same genetic or environmental components. Strong genetic association with ASD is supported by data from high monozygotic twin concordance and large-scale genetic screens have revealed numerous autism risk factors [7], each with relatively low penetrance. This supports the hypothesis that the behavioral manifestations of ASD may be a consequence of genetic or environmental factors. A recent report showed a higher concordance among dizygotic twins that generates a best-fit model that attributes a 55% contribution of environmental factors and a 37% contribution of genetic factors as high risk for autism [8]. 

Environmental insults leading to ASD occur early in gestation during first or second trimester [9,10]. Prenatally exposed children to the anticonvulsant agent valproic acid early in the first trimester of gestation, significantly increased the incidence of autism in children [11]. Approximately 5% of individuals exposed *in-utero* to thalidomide between day 24 and 36 of gestation [12], as well as misoprostol [8], developed ASD. Several studies have suggested that maternal infections associated with fever [13,14], immune activation [15,16], significant bleeding during the second trimester [17] or occurrences of cytomegalovirus infection during the third trimesters [18] are risk factors leading to ASD. Other factors that have been linked with increased risk of ASD in offspring include older paternal age, prenatal stress, maternal diabetes and obesity [19]. These reports are consistent with the reports that autism is associated with impaired brain development [20] that affects the amygdala, cerebellum and many other regions of the brain [21]. The brains of children with autism tend to grow faster than usual just after birth, followed by normal or relatively slower growth in childhood and ASD involves specific regions of the brain [22].

Although extensive studies have been carried out on autism, there are still controversies regarding its underlying mechanisms. Evidence of neurodegeneration has been seen in some cases of children with ASD who experienced progressive loss of neurological function, in the form of activated microglia and astrocytes, elevated 8-oxoguanine levels, evidence of oxidative stress, the presence of pro-inflammatory cytokines and neuronal cell loss [23]. The World Health Organization considers ASD to be a developmental disorder exclusively affected by environmental factors and genetics, rather than mere neurodegeneration [23]. 

In addition, the human immune cellular and genetic components rely on environmental exposures to induce the expression of genetically encoded signaling influencing effector functions. The maternal immune system control and regulate the health of both mother and fetus. Thus, the components of the maternal immune system that cross the placenta may be considered to be fetal environmental exposures. However, proper fetal neurodevelopment relies on the precise timing, functional levels and anatomic localization of many signaling molecules that may be altered by exogenous factors to which the embryo is exposed [24]. 

We, therefore, carried out a study to detect the presence of IgG class circulating autoantibodies against neuronal and glial proteins in the sera of 40 subjects (10 children with ASD and their mothers and 10 control children and their mothers as controls). We determined autoantibodies against neural proteins that are associated with neurogenesis (NFP, tubulin, tau, MAP-2 and alpha-syncline), myelino genesis (MBP and MAG) and astrogliogenesis (GFAP and S100B, both of which are secreted by astrocytes).

## 2. Materials and Methods

### 2.1. Subjects

Under the protocol approved by the Institutional Review Board of Duke University Medical Center, serum samples were collected from a total of 40 subjects as follows: 10 subjects in each of the following four groups: children with ASD, mothers of children with ASD, control children and mothers of control children. The control children and their mothers did not have any signs or symptoms of ASD. The mothers of both the groups were age-matched and the children (ASD & Control) were matched for the gender and age. Informed consent for blood collection was obtained from the parents of all children enrolled in this study, in accordance with the Code of Ethical Principles for Medical Research with Human Subjects of the World Medical Association. Assays were performed within a few days of obtaining the blood samples and the sera were frozen and stored at −80 °C.

### 2.2. Diagnostic Criteria

Prior to entering the study, subjects were administered, the Autism Diagnostic Interview-Revised version, the Childhood Scale Scoring (CARS) (a minimum score of at least 30 points was required to be included in the study) and the Autism Diagnostic Observation Schedule-Generic in order to document the diagnosis of autism [25]. The Diagnostic and Statistical Mental Disorders-5 (DSM-5) autism spectrum disorder criteria were applied to all patients. 

### 2.3. Exclusion Criteria

Potential subjects were excluded if they had any of the following: a neurological or comorbid psychiatric disorder, epilepsy, a history of liver dysfunction, renal or endocrine disorders, or any kind of current infection. Intellectual disabilities or behavioral disorders, including generalized developmental disorders (PDD), not otherwise specified (PDD-NOS) and attention deficit hyperactivity disorders were also considered as exclusion criteria. Children diagnosed with Asperger’s Syndrome (although included in DSM-5), Fragile X Syndrome and Tuberous Sclerosis were also excluded from the study. Other exclusion criteria were celiac disease and/or other major diseases of the intestinal tract, such as inflammatory bowel disease or liver disorders. The IQ test was not performed. The subjects did not receive any pharmacological intervention. 

### 2.4. Therapeutic Treatments

Six out of 10 patients were on a gluten/casein-free diet and 10 patients were treated with vitamins and minerals. One patient has memantine as an off-label treatment. None of the patients had other known medical or psychological conditions. All healthy patients and controls were free of infections, fever or other medical conditions at the time of blood collection and did not take steroids or NSAIDs for at least 30 days prior to blood collection.

Power analysis for a number of subjects and controls was performed based on a t-test of two samples, assuming a common standard deviation between the groups. The power analysis was parameterized by the Cohen d, which measures the size of the effect as the ratio of the difference in the means of the group with a common standard deviation. In practice, a well-fed study would be able to achieve 80% power for lower Cohen’s d values. Based on our power analysis, a 1:1 study design would be propelled to 80%. For this reason, we have chosen to go with a 1:1 design (10 ASD children and their mothers and 10 normally developing control children and their mothers).

## 3. Demographic Information

### 3.1. Subjects

The ASD children consisted of two boys and eight girls and the same proportion was among the control children. Their ages at the time diagnosis of autism ranged from 2 to 4 (mean: 3.4) years. Their ages at blood collection ranged from 3 to 18 (mean of 8.40) years compared to 6 to 12 (mean: 8.21) years for healthy children who served as controls.

### 3.2. Mothers

The ages of the mothers of ASD children at the time of the birth of these children ranged between 31 and 56 years with the mean age 35.41 years and mothers of control children ranged from 31 and 43 with the mean age of 36.32 years. At the time of blood donation, the mothers of ASD children had ages that ranged from 38 to 58 (mean: 43.2 years) compared to age of the mothers of healthy developing children 34–47 (mean: 39.43) years.

### 3.3. Parental Education

Among the mothers of ASD children, two had a secondary education, while the other eight had a college education. On the other hand, among healthy control mothers, five had a high school diploma and five had a college education.

### 3.4. Signs of ASD in Study Children

Signs of the first indications of ASD were the loss of language or social skills (9 children), followed by no sound or social response and no response to names (7 children each) and the details are presented in Table 1. Signs of later indicators include an impaired ability to make friends and stereotyped behavior, repetitive or unusual use of language (10 children each), followed by decreased ability to initiate or maintain a conversation with other children and impaired social abilities and imaginative play (8 children each).

## 4. Materials

The sources of protein were: Recombinant human proteins NFP, tau, MAP-2, tubulin and MBP purchased from Sigma-Aldrich (Sigma-Aldrich Corporation, Saint Louis, MI, USA); human recombinant GFAP from Biotrend Chemikalien GmbH (BIOTREND Chemikalien GmbH, Cologne, Germany); MAG recombinant protein from Noves Biologicals; S100B from American Qualex International, Inc. (American Qualex International Inc., San Clemente, CA, USA); α-Synuclein from Boston Biochem; human IgG from Fitzgerald; Goat anti-human IgG conjugated to horseradish peroxidase and the enhanced chemiluminescence reagent were obtained from Amersham Pharmacia Biotech (Amersham Pharmacia Biotech, Piscataway, NJ, USA). SDS-PAGE gels (4–20% gradient gel) and 15 mM tris-glycine were obtained from Invitrogen (Invitrogen, Carlsbad, CA, USA). All other buffers were purchased from Thermofisher/Bio-Rad (Thermo Fisher Scientific, Waltham, MA, USA), USA (Table 2 provides molecular weights and protein location in the brain;16).

### 4.1. Western Blot Assay

Western blot analysis was used to determine human sera autoantibodies binding to specific proteins in ASD, controls and their mothers. This assay allowed the determination of the autoantibodies against major proteins and their isoforms (see Table 2 for the molecular weight of each peptide detected). Each serum sample was analyzed in triplicate. All proteins were loaded at 100 ng/lane. IgG is used as an internal control for each of the sera tested. The proteins were denatured and electrophoresed on SDS-PAGE (4–20% gradient gel) purchased from Invitrogen (Carlsbad, CA, USA). The proteins were transferred to polyvinylidene fluoride (PVDF) membranes (Amersham). Nonspecific binding sites were blocked with Tris-buffered Saline-Tween (TBS-T) (40 mM Tris (pH 7.6), 300 mM NaCl and 0.1% Tween 20) containing 5% fat-free milk powder for 1 hour at 22 °C. Membranes were incubated with serum samples at 1: 100 dilutions in TBS-T with 3% nonfat milk powder overnight at 4 °C. After five washes in TBS-T, the membranes were incubated with a 1:2000 dilution of goat anti-human IgG conjugated to horseradish peroxidase (Amersham). Membranes were developed by enhanced chemiluminescence using the manufacturer’s protocol (Amersham) and developed using a Typhoon 8600 variable model recorder (GE). The signal intensity of each lane was quantified using Bio-Rad image analysis software (Hercules, CA, USA). Periodically the PVDF membranes were stained with Coomassie blue to check the protein transfer efficiency. All tests were performed in a blinded fashion.

### 4.2. Specificity of Serum Autoantibodies

To demonstrate the specificity, selectivity and reproducibility of our assay, we used competition assays, ensuring that the sera containing autoantibodies can bind to the target antigen (using the purified proteins). The specificity of the serum autoantibody against all tested neural proteins was carried out by performing a protein/peptide absorption assay. Previously, we have checked the specificity of the serum autoantibody levels by performing an absorption assay, where the serum was preincubated with the target protein or peptide [26]. In this study, we used the serum from ASD children and control children, where we incubated the sera overnight at 4 °C with S100B, GFAP, tau, MAP2, NFP, tubulin, MBP SNCA or MAG. The protein mix was centrifuged at 15,000 rpm to deplete any immune complexes. The supernatants were then carefully removed and used in Western blotting.

### 4.3. Calculations

The signal intensity of each lane was quantified as optical density measurements for subjects and controls. The values obtained were divided by the concentration of serum IgG; this value for each subject was normalized to controls and expressed as change from healthy controls. The results are expressed as mean of triplicate assay values of arbitrary optical density units normalized to the optical density obtained using IgG (internal control), as compared to healthy controls.

## 5. Statistics

The pooled data are presented as mean ± SE. Subjects’ values were compared to the control group using a paired t-test. Mean values of the group of subjects within the groups were compared using one-way ANOVA (Sigma Stat; Software Systat) and a p-value < 0.05 was accepted as statistically significant.

## 6. Results

**Figure 1:**
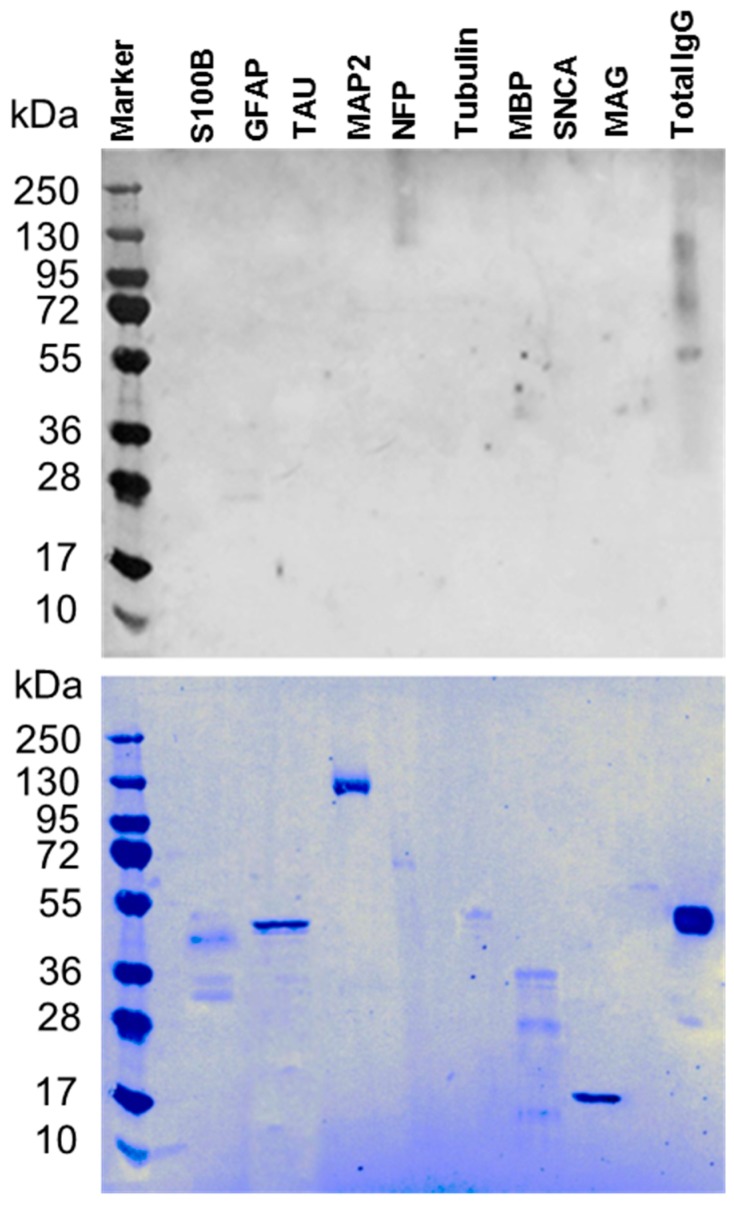
Specificity of the serum autoantibody. The specificity of the serum autoantibody against all tested neural proteins was carried out by protein/peptide competitive or absorption assay. Upper panel) Western blot from ASD-C following the absorption of the sera with S100B, GFAP, Tau, MAP2, NFP, Tubulin, MBP, SNCA and MAG proteins. The levels of autoantibodies against neuronal and glial proteins are negligible supporting the specificity of the assay. Lower panel) Commassie stain showing the proteins signals on the blot indicating that the sera were depleted from the autoantibodies by the adsorption reaction.

This study determined the level of autoantibodies against a panel of 9 neuronal proteins reported to have low levels in autistic patients higher levels in children with ASD compared to lower levels in control children [24,27]. Sera were collected from 40 subjects (10 children with ASD and their mothers compared with the 10 control children, age-matched and their mothers, as controls). Sera were analyzed by western blot analysis. The specificity of autoantibody in the sera was assessed by performing peptide/antigen absorption assay by preabsorbing the serum with the target proteins [26]. The preabsorbed serum of ASD patient was tested by western blot (Figure 1). The lack of specific bands for the neuronal proteins confirmed the specificity of the autoantibodies in the sera. Coomassie staining of the membrane confirmed the loading efficiency of these proteins.

### 6.1. Autoantibodies against Neuronal and Glial Proteins

**Figure 2:**
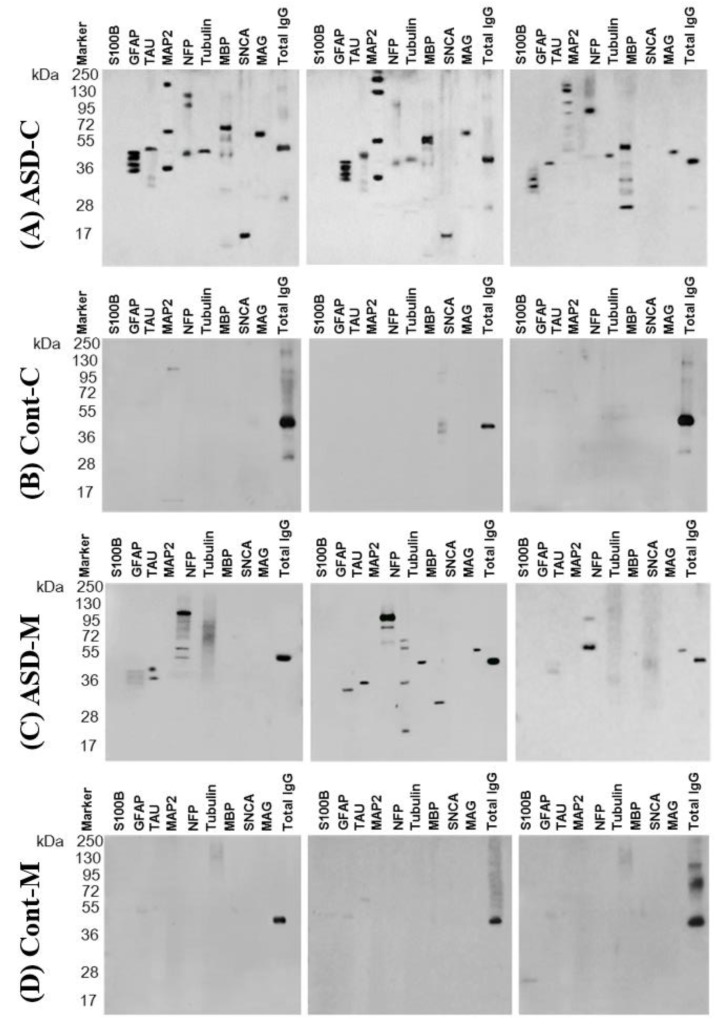
Representative panel of Western blotting from three cases of the (ASD-C)—ASD children (**A**), (Cont-C)—control children (**B**), (ASD-M)—mothers of ASD children (**C**) and (Cont-M) the mothers of control children (**D**) are shown with the molecular weight marker. Total IgG was used as a reference protein. Note that the IgG fractionates as 150 kDa on a native gel consisting of an intact IgG molecule, however, when fractionated on a denaturing gel, it resolves as two heavy (H) chains and two light (L) chains showing thick bands at 50 kDa and light bands at 25 kDa. Bands in each lane were quantified on digitized images in the mid-dynamic range and normalized to the value of IgGband density.

We checked for the presence of autoantibodies in the sera collected from the four groups of ASD children and their mothers and healthy control children and their mothers. Figure 2 shows representative micrographs of a western blot for sera from ASD children (Figure 2A) and their mothers (Figure 2C). The sera reacted intensely to neural proteins, while most of the control children (Figure 2B) and their mothers’ sera Figure 2D) showed a week or no reaction to the panel of proteins. 

### 6.2. Levels of Autoantibodies against Neuronal and Glial Proteins in ASD Children and Their Mothers

**Figure 3:**
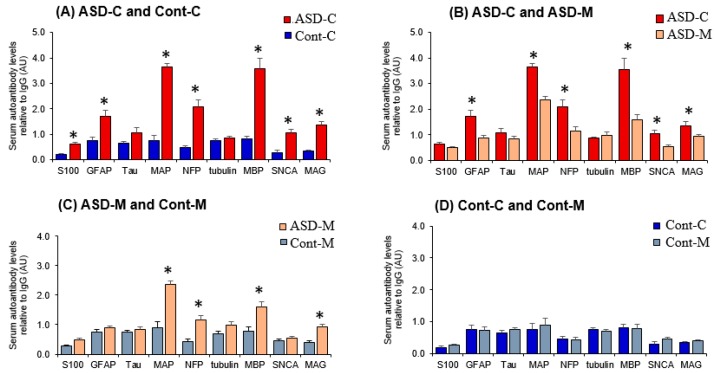
Histogram representing the mean levels of autoantibodies against neural proteins relative to total IgG with the bar indicating standard error. Comparison between (**A**) control children (Cont-C) and ASD children (ASD-C); (**B**) ASD Children (ASD-C) and ASD children’s mothers (ASD-M); (**C**) ASD children’s mother (ASD-M); (**D**) control children (Cont-C) and control children’s mothers (Cont-M). Significant differences with P < 0.05 are indicated in asterisks and the actual p-values are presented in Table 3.

The quantitative analysis of the reactivity of sera from ASD children showed significantly elevated levels of circulating IgG class autoantibodies against the nine proteins analyzed compared to controls children (Figure 3A, Table 2). The mean levels of autoantibodies in subjects and controls were in descending order: anti-MAP-2 > anti-GFAP > anti-MBP > anti-NFP > anti-tau > anti-MAG > anti-SNCA > anti-Tubulin> and anti-S100B. The sera from the mothers of ASD children exhibited high levels of autoantibodies in the order of MAP > MBP > NFP > GFAP > MAG > SNCA and tau, but not for S100B and tubulin compared to the sera from the mothers of control children (Figure 3B, Table 2). The autoantibodies compared between mothers of ASD children and control children showed a significant increase in the order of MAP > MBP > NFP > MAG (Figure 3C, Table 2). The Sera from healthy control children and their mothers showed none to very low reactivity to the tested proteins (Figure 3D, Table 2).

The results are expressed as mean ± SEM. Values of triplicate assays of optical density arbitrary units normalized to the optical density of IgG. Statistical differences are obtained by Two-way ANOVA followed by pairwise multiple comparison procedures (Tukey Test). The pairwise comparison was done between ASD children and the control children and the significance is represented in asterisks (*) in the relevant values in the column consisting of ASD children. A similar comparison was performed between the mother’s group and the significance is represented by asterisks (†) in the column containing mothers of ASD children.

### 6.3. Autoantibodies Profile of Neuronal and Glial Proteins in ASD and Control Children and Their Mothers

**Figure 4:**
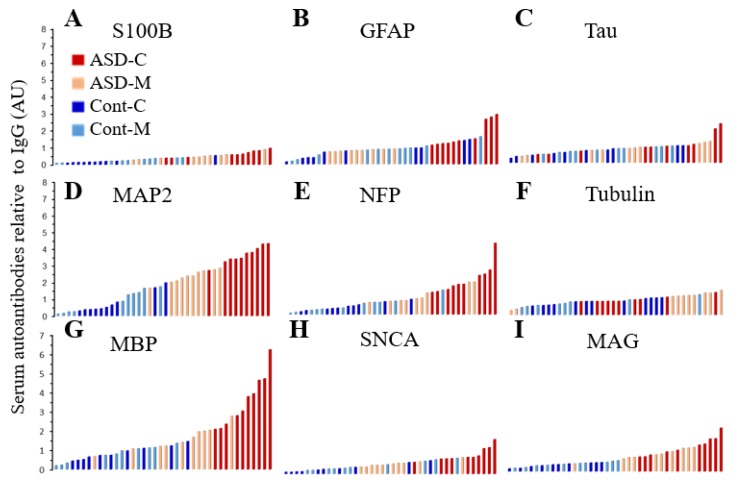
Histogram representing the profiles of individual levels of serum autoantibodies in ASD children (ASD-C) represented in red color bars; ASD children’s mothers (ASD-M) represented in light rose color bars; Control children (Cont-C) represented in royal blue color bars and Control children’s mothers (Cont-M) represented in light blue color bars. (**A**) S100B; (**B**) GFAP; (**C**) Tau; (**D**) MAP2; (**E**) NFP; (**F**) Tubulin; (**G**) MBP; (**H**) SNCA and (**I**) MAG.

An array of autoantibody profile tested for a panel of nine neuronal and glial proteins each representing individual values are presented as four groupsin ascending order can be easily visualized in Figure 4. The sera from ASD children contain the highest fold increase for anti-MAP2, anti-MBP, anti-NFP and anti-MAG.

### 6.4. Fold Increase in Autoantibodies against Neuronal and Glial Proteins in ASD Children and Their Mothers

Quantitation of the autoantibodies against the panel of nine neuronal and glial proteins are presented in the Table 3 as fold increase calculated from the values of healthy control. The sera from ASD children contain the highest fold increase in the order of anti-SNCA > anti-MAP2 > anti-NFP > anti-GFAP > anti-MBP and can be correlated with the anti-MAP2 and anti-NFP in their mothers (Table 4). Furthermore, the fold increase reflected the highest P-value when compared for MAP2 and NFP (P < 001). The comparative data is presented in Table 4.

The results of fold increase for each neural autoantibody in the serum of children with ASD compared to control and comparison of their mothers are expressed as mean ± SEM. Statistical comparison was performed by Two-way ANOVA followed by pairwise multiple comparison using Tukey Test.

### 6.5. Distribution of the Biomarkers for ASD Subjects and Controls

**Figure 5:**
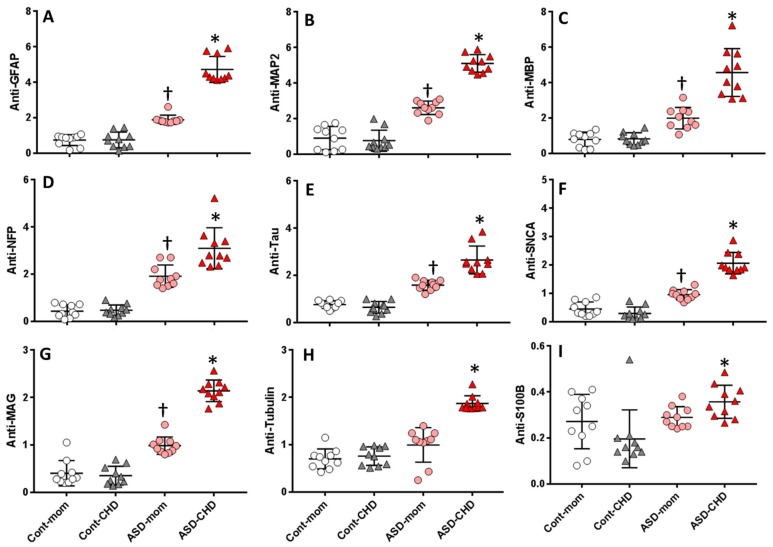
Scatter Plot showing the distribution of neural autoantibodies in Control Children’s mothers (Cont-M), Control children (Cont-C), ASD children’s mothers (ASD-M), ASD children (ASD-C) relative to the total IgG levels. Autoantibodies against (**A**) GFAP. (**B**) MAP2, (**C**) MBP, (**D**) NFP, (**E**) Tau, (**F**) SNCA, (**G**) MAG, (**H**) Tubulinand, (**I**) S100B. The cross line on the dot plot represents the mean values for each protein. The vertical line represents SEM. ^†^ P > 0.05 or * P < 0.01 is considered significant.

In order to determine the pattern of individual profiles of autoantibodies to each neuronal protein, a comparative scatter blot was done between four groups to distinguish the levels of their profiles and to determine the degree of autoantibody distribution. An autoantibody level to each neuronal protein was separately compared between individual cases among four groups for distribution among groups and within groups (Figure 5A–I). A distinct distribution was seen in ASD children sera for anti-S100B, anti-MAP2, anti-MBP, anti-NFP, anti-SCNA and anti-MAG with no overlap within or among groups (P < 0.001) compared to some overlap observed for anti-GFAP, anti-Tauandanti-tubulin within groups. Comparison between neuronal protein autoantibodies in the sera of ASD children and sera from their mother showed distinctly elevated levels of autoantibodies against all nine proteins and the profile overlap was observed in anti-NFP, anti-MBP, anti-S100B and anti-tau. Similar analyses were done to compare the autoantibody levels to the neuronal proteins in the sera of control children to that of their mothers. Though the levels of autoantibody were low and there was overlap within groups for all proteins but there were no distinguishable differences observed. The comparison of the mothers of control children and the mothers of ASD children clearly show an elevated distinct distribution in the sera from the mothers of ASD children and the visual contrast was observed in anti-MAP2, anti-GFAP, anti-tau, anti-NFP and anti-MAG. 

This study showed that autoantibodies to MAP2, MBP and NFP were significantly elevated in the sera of ASD children, although the same trend was seen in MAG, tubulin and tau the relative autoantibody level was significantly lower compared to MAP2, MBP and NFP. The astroglial marker S100B which is usually expressed in myelinating and non-myelinating Schwann cells seems to be the lowest in detection, yet the overall comparison showed significantly elevated levels in ASD children but not their mothers. Overall the scatter distribution of autoantibody levels to each of the neuronal and glial proteins gives a visual comparison of the distribution of the levels among the study groups.

## 7. Discussion

Despite the obvious small sample number of subjects in this preliminary study, the robust similarity between ASD children and their mothers and control children and their mothers further affirm the significance and contribution of maternal autoantibodies being one of the major factors in developing ASD. The higher levels of MAP-2, NFP, MBP, MAG, α-synuclein S100B and GFAP (3.2 to 4.8-fold) autoantibodies reported in ASD children warrant further research for developing autoantibodies screening as a biomarker for detection of early autism. Autoantibodies have been used as biomarkers for brain injury owing to chemical exposures and have been validated in several studies from our laboratory. Serum autoantibodies of IgG class from the mothers can cross the placenta, which may alter brain development [28]. Although the exact role of these autoantibodies in the development of ASD is not known, it is thought that they may affect key neurons in the cerebellum by binding to and altering their physiological role [29].

The study reports a significant elevation of the autoantibodies against neuronal and glial proteins in the serum of ASD children and their mothers, compared to age-matched control children and their mothers. The results of this study suggest that the profile of the peripheral autoantibodies against the neural proteins is unique to ASD children as compared to the profiles found in other brain disorders [30]. Our previous studies documented the presence of IgG autoantibodies against neuronal and glial proteins in sera of aircrews and farm workers who were exposed to organophosphates (OP) and developed OP-induced delayed neurotoxicity(OPIDN) as well as the following exposure to molds [26,31,32]. A recent study from another laboratory-confirmed our finding in another cohort of aircrews using Magnetic Resonance Imaging (MRI) examination [33]. The MRI results showed deficits in white matter brain microstructures and cerebral perfusion that are potentially causative to cognitive impairments and mood deficits reported by aircrew [33]. The extent of cognitive impairment was strongly associated with white matter integrity. These results are consistent with the present results where there are increased autoantibody levels against MBP, MAG and NFP reflecting, myelin degeneration. ASD is characterized by increased brain volume and in some cases, there is an altered ratio of gray/white matter. The affected areas of the brain include the cerebellum, cortex, nuclei of the amygdala, the fusiform face area and parts of the limbic system [34]. Brain abnormalities in ASD include excess white matter neurons and decreased numbers of cerebellar Purkinje cells [35,36]. Axonal transport is an essential process for the continuous delivery of materials from the cell body to the nerve terminal. Protein components of the axonal transport include neurofilament triplet proteins (NFP), which is also associated with maturating brain, tubulin and microtubule-associated proteins, MAP-2 and tau [37]. Neuronal tau proteins are more abundant in white matter than gray matter and are elevated in cerebrospinal fluid (CSF) and serum after traumatic brain injury (TBI) [38]. Aggregation of Tau has been reported and used as a diagnostic marker for Alzheimer’s disease [39]. Myelinated axons contain myelin basic protein (MBP) and myelin-associated glycoprotein (MAG). MBP may aid in the clinical evaluation of multiple sclerosis and stroke [40]. The most abundant microtubule-associated protein in the mammalian brain is MAP-2, found in the dendrite-rich Purkinje cell. α-synuclein functions as a neuroprotective protein against oxidative stress [41]. The GFAP astrocytic protein contributes to white matter architecture, myelination and integrity of the blood-brain barrier [42]. The S100B, another astrocytic protein interacts with and stabilizes proteins associated with microtubules, such as tau and MAP-2 and exerts deleterious effects on neutrophils, depending on its concentration in the brain tissues [43]. 

The results show that MAP-2 autoantibody levels were the highest that is consistent with reports showing the lower density of Purkinje cells coupled with decreased dendritic MAP-2 neurons in ASD children [44,45]. Loss of MAP-2 is a reliable indication of irreversible neuropathological alterations, such as brain damage [46]. The increase of autoantibodies against neurofilaments (NFP) in ASD subjects is consistent with the destruction of neurofilaments in neurodegeneration [47]. Also, the presence of NFP in cerebrospinal fluid (CSF) and serum has been suggested to reflect the destruction of axons and rupture of the BBB further causing axonal injury [48]. Autoantibodies to MBP showed the next highest levels in ASD subjects compared to control children. These results are also in agreement with an increase of autoantibodies against the myelin-associated glycoprotein protein MAG and the involvement of white matter in autism [40]. 

Although autoantibodies to astrocyte proteins increased significantly in ASD children compared to controls, they were lower than those in neuronal proteins. GFAP and S100B may reflect specific CNS changes and axonal damage, thus serving as biomarkers for ASD and that serum concentrations of autoantibodies against specific brain proteins obtained from children with ASD are related to the severity contributing to the deficit associated with this disorder. The action of S100B is related to its serum concentration, it is at a nanomolar level at the onset of the injury and the concentration is at a micromolar level during apoptotic phase [43]. Acute traumatic brain injury resulting in large destruction of astrocytes leads to a massive (50- to 100-fold) release of S100B in serum, whereas levels of S100B in psychiatric disorders were only three times higher in patients compared to the controls [49], correlating well with their neuroprotective ability in the present study. These findings have been documented in cases of dementia, particularly Alzheimer’s disease, schizophrenia and major depression [50] and mania [51]. It has been hypothesized that the serum concentration of S100B may also be a predictive value of the autistic brain. However, because of its short half-life in serum, approximately 2 hours [43,52], its assessment is limited for short-term acute brain injury cases.

The role of the immune system in the development of ASD has been explained by dysregulation of immune function [53], neuroinflammation [54] and maternal autoantibodies [55]. The present results agree with previous studies that detected serum autoantibodies against some human unidentified brain proteins using ELISA and Western immunoblot in children with ASD and their non-autistic siblings [14,24,55,56,57,58,59,60,61,62,63,64,65,66,67,68,69] and [24,56,57,64,65,69]. Although these studies did not specifically identify these proteins, we can extrapolate based on their reported masses, that some were probably neuronal and glial proteins. We have listed in Table 5, how the masses of these unidentified proteins, can likely correspond to masses of known neuronal and glial proteins that we have used in our study, with the most notable identical matches being MBP, tau, tubulin, NFL and MAG.

Our results are consistent with the opinion that maternal neuronal cell death could contribute to the formation of autoantibodies against neuronal and glial proteins. Further, modern synthetic pesticides are usually neurotoxicants that are designed to target the nervous system [58], and recent studies, confirmed that the pesticide exposure can cause neuronal cell death [30,31,32]. Also, studies reported the development of autoantibodies against neuronal and glial proteins in patients exposed to pesticides who developed neurological symptoms characteristic of those caused by the insecticides [31]. Epidemiological evidence showed that mothers exposed to pesticides near conception increased their likelihood of having children with ASD [59]. Living near agricultural fields, ASD has been linked to the following pesticides: organophosphorus (OP) insecticides such as diazinon and chlorpyrifos [60,61], the OP herbicide glyphosate [62], pyrethroid insecticides [19] and the organochlorine pesticides dicofol and endosulfan [63].Our present study supports the assumption that ASD may be an autoimmune disease that involves maternal perturbations in B-cell development and subsequent formation of IgG autoantibodies against neuronal and glia-specific proteins.

## 8. Limitations

Although the present pilot study can serve as a proof-of-concept, the sample in this study was too small to examine important covariates such as the mothers’ age, lifestyle and exposure to environmental factors. Although no information is available on the exact mechanism of autism, the present study supports the assumption that ASD may be an autoimmune disease that may include maternal IgG autoantibodies and provides peripheral markers for the diagnosis and/or confirmation of ASD. When the balance between B-cell activating and inhibitory signals is disturbed, it becomes predisposed to produce pathogenic autoantibodies (IgG) and autoimmunity. Perturbations in B-cell development gives rise to autoantibodies that are the hallmark response to immune reactions against self-tissues [70]. If the results of the current study are confirmed in a larger number of patient cohorts and controls, therapeutic intervention could take place by impairing protective immunity of B cells [71]. A major strategy for the treatment of human autoimmune diseases is using less toxic therapies that render normal B cell’s function normal by eliminating the pathogenic autoantibodies. Rituximab (Rituxan) has been effectively used to reduce the number of B cells, without causing toxicity [72].

## Figures and Tables

**Table 1:** Early and late signs of Autism in children included in this study.

	**Early Indicators**	**YES**	**NO**
1	No babbling or pointing by age 2	2	8
2	No single words by 16 months or two-word phrases by age 2	6	4
3	No response to name	7	3
4	loss of language or social skills	9	1
5	Poor eye contact	5	5
6	Excessive lining up of toys or object	3	7
7	No smiling or social responsiveness.	7	3
	**Later indicators**	**YES**	**NO**
1	Impaired ability to make friends with peers	10	0
2	Impaired ability to initiate or sustain a conversation with others	8	2
3	Absence or impairment of imaginative and social play	8	2
4	Serotyped, repetitive, or unusual use of language	10	0
5	Restricted patterns of interest that are abnormal in intensity or focus	4	6
6	Preoccupation with certain objects or subjects	4	6

**Table 2:** Glial proteins (Mass and location).

Neural Protein	Mass kDa	Location in the Brain
NFL, NFM, NFH	70,160, 200	Axon is mainly found in large myelinated axons
Tubulin	55	Axon consists of 10–20% of soluble protein in the brain, axonal transport
Tau	45–62	Axon, More abundant in the white matter
SNCA	14	Axon, a neuroprotective protein, with respect to oxidative stress
MAP2	200	Dendrites, Purkinje cells, Cerebellum
MBP	38	Myelin
MAG	100	Myelin, near the axon
GFAP	52	Astrocytes, contribute to white matter, myelination and BBB. forms Glial Scar,
S100B	10–11	Astrocytes, neurotropic at nanomolar concentration and apoptotic at micromolar

**Table 3:** Autoantibodies against neuronal proteins in the sera of children with autism (ASD-C), healthy children (Cont-C), mothers of ASD children (ASD-M) and mothers of healthy children (Cont-M).

Protein Name	ASD-C	Cont.-C	ASD-M	Cont.-M	P Value
S100B	0.36 ± 0.06 *	0.20 ± 0.04	0.29 ± 0.06	0.27 ± 0.04	0.022
GFAP	4.72 ± 0.23 *	0.75 ± 0.14	1.88 ± 0.08 ^†^	0.74 ± 0.10	0.001
Tau	2.63 ± 0.19 *	0.65 ± 0.08	1.58 ± 0.09	0.76 ± 0.05	0.005
MAP2	5.09 ± 0.16 *	0.76 ± 0.19	2.84 ± 0.12 ^†^	0.91 ± 0.21	0.001
NFP	3.10 ± 0.28 *	0.47 ± 0.07	1.92 ± 0.15 ^†^	0.44 ± 0.08	0.001
Tubulin	1.87 ± 0.05 *	0.76 ± 0.06	0.99 ± 0.12	0.70 ± 0.07	0.043
MBP	4.56 ± 0.43 *	0.83 ± 0.11	1.89 ± 0.18 ^†^	0.79 ± 0.13	0.001
SNCA	2.06 ± 0.12 *	0.30 ± 0.08	0.95 ± 0.05	0.45 ± 0.08	0.001
MAG	2.14 ± 0.16 *	0.35 ± 0.04	0.99 ± 0.08 ^†^	0.40 ± 0.04	0.001

The results are fold increase of neural serum autoantibodies compared to controls and expressed as mean ± SEM. * denote significance. A similar comparison was performed between the mother’s group and the significance is represented by asterisks (^†^) in the column containing mothers of ASD children.

**Table 4:** Fold Increase of autoantibodies against neural proteins in the serum of children with ASD compared to control children. Similar comparisons were made between mothers of ASD children and mothers of control children.

Protein	ASD-C Over Cont-C	ASD-M Over Cont-M	P-Value
S100B	1.83 ± 0.31 *	1.07 ± 0.21	0.022
GFAP	6.29 ± 0.31 *	2.54 ± 0.11 *	0.005
Tau	4.08 ± 0.29 *	2.08 ± 0.12 *	0.005
MAP2	6.70 ± 0.21 *	3.13 ± 0.13 *	0.001
NFP	6.57 ± 0.55 *	4.39 ± 0.33 *	0.001
tubulin	2.46 ± 0.07 *	1.41 ± 0.17	0.025
MBP	5.51 ± 0.71 *	2.38 ± 0.24 *	0.026
SNCA	6.99 ± 0.40 *	2.13 ± 0.11 *	0.036
MAG	6.05 ± 0.54 *	2.47 ± 0.20 *	0.001

The results are fold increase of neural serum autoantibodies compared to controls and expressed as mean ± SEM. Statistical differences is obtained by Two-way Anova followed by pairwise multiple comparison procedures (Tukey Test). * denote significance.

**Table 5:** Assignment of neural protein identity by molecular mass (in kDa).

Protein Mass (kDa)	Corresponding Neural Protein	Location in the Brain	References
10–11	S100B	Brain glia and astrocytes	[68]
14	SNCA	neocortex, hippocampus, substantia nigra, thalamus, cerebellum	[14]
36	MBP	Cerebellum	[64]
37	MBP	Cerebellum	[24,55,56,57]
39	MBP	Cerebellum	[24,55,56,57]
45–62	Tau	Cerebellum	[24,59,65]
200	MAP2	Brain	[66]
52–55	Tubulin	Cerebellum	[69]
67–71	MAG	Cerebellum	[24,56]
70–150	NFP	caudate, putamen and prefrontal cortex	[56,57,64]

## Abbreviations

ASD	Autism Spectrum Disorder
TD	Typically Developing
DD	Developmental Delay
ASD-C	Children with Autism Spectrum Disorder
ASD-M	Mothers of ASD children
Cont-C	Control healthy Children
Cont.-M	Mothers of control healthy children
AU	Arbitrary Unit
